# Eudaimonia as Personal Growth: Decades of Research and Needed Future Directions

**DOI:** 10.3390/bs16060966

**Published:** 2026-06-11

**Authors:** Carol D. Ryff

**Affiliations:** Department of Psychology & Institute on Aging, University of Wisconsin-Madison, Madison, WI 53706, USA; cryff@wisc.edu

**Keywords:** personal growth, eudaimonia, demographic factors, health outcomes, inequality, vital inputs, virtue and ethics

## Abstract

This article examined a conception of personal growth put forth in a multidimensional model of psychological well-being over 35 years ago. The model is widely used around the world. One key dimension of well-being is personal growth, which is examined in detail here. The conceptual underpinnings of this construct are reviewed, as well as its formal definition and empirical operationalization. Decades of scientific research about personal growth are selectively reviewed, focusing on factors that predict different levels of personal growth as well as how it is linked with other phenomena such as health, biological risk factors, and cognitive capacities. Future directions in the science and practice of personal growth are put forth, including the need to address obstacles to personal growth tied to widening inequality, the role of the arts and humanities in nurturing personal becoming, and the need to elevate virtue and ethics as core features of eudaimonia, with exemplars from human history provided.

## 1. Introduction

This article provides an in-depth look at a particular conception of personal growth and its scientific impact. The first section examines conceptual foundations of personal growth, a key dimension in a multidimensional model of psychological well-being (Ryff, 1989). The objective is to revisit how various scholars articulated what it means to experience personal growth as well as how these differing formulations were integrated into an overarching perspective. Attention is also given to self-report scales constructed to assess the degree to which individuals view themselves as experiencing personal growth. The second part then selectively examines some of the scientific research on factors that predict different levels of personal growth and links it to other phenomena, such as health, biological risk factors, and cognitive capacities. A final section focuses on needed future directions in the science and practice of personal growth, giving emphasis to three primary topics: (1) obstacles to personal becoming, especially widening inequality, (2) the role of the arts and humanities in nurturing self-realization, and (3) the need to elevate virtue and ethics as core features of eudaimonia as articulated by Aristotle over 2000 years ago.

## 2. Conceptualizing Personal Growth

From the middle of the last century to the present, numerous scholars have engaged with the topic of personal growth, using a variety of different terms. These warrant revisiting to clarify various ways in which this elusive concept has been articulated. What follows below first identifies these distant writings, seeking to distill the meanings they gave to what it means to be self-realized and fully functioning. The next section then integrates these multiple formulations and offers a distilled definition of personal growth from which a self-report scale was constructed so as to measure individuals’ perceptions of the degree to which they see themselves as growing and making the most of their talents and capacities over time.

### 2.1. Varieties of the Growth Experience

Forty years ago, personal growth was examined as a significant component of motivation in adulthood and aging (Ryff, 1985). In contrast to outward life achievements, such as salary, educational status, and occupational prestige, the focus in personal growth is on internal experiences, such as striving for growth, self-awareness, and personal insight. Multiple prior formulations seeking to distill the components of personal development were examined, while paying attention to how the authors came up with these formulations. Who did they study in trying to articulate the meanings of growth? What did they offer as explanations for the process of becoming?

Maslow (1955, 1968) formulated self-actualization to provide an alternative conception of human experience compared to mechanistic views in behavioral psychology, or depth views in psychoanalytic psychology. In his view, both of these saw the individual as at the mercy of external circumstances or uncontrollable internal forces. He wanted to advance knowledge of the best that individuals could become and thus studied well-developed individuals, choosing from among his friends, associates, and historical figures. The goal was to identify common qualities in such individuals. He believed that the need to grow and develop is present in all at birth. That is, he saw the motivation to grow as universal and instinctive.

Maslow organized human motivation into a hierarchy of needs. Lower needs, such as physiological requirements and safety, must be satisfied before moving to self-actualization, which involves a higher need for self-fulfillment and complete development of one’s capacities. He saw self-actualizers as moving toward meta-needs (beyond deficiency- or being-needs), which included truth, goodness, beauty, unity, wholeness, justice, and order. Maslow detailed many characteristics of self-actualizers, including their character structure, humor, creativity, independence, naturalness, spontaneity, freshness of appreciation, and deep interpersonal relationships. He also noted they sometimes experienced intense or overwhelming ecstasy (mystical or peak experiences).

Rogers (1961) described the fully functioning person, drawing on his work with individuals in therapy. His client-centered approach tried to help people become more aware of their inner selves, more accepting, and capable of dealing with distress. He believed that individuals have within themselves the tendency to move toward maturity, i.e., to expand, extend, develop, and express all of their capacities. This innate motivation encompassed both physiological and psychological components of growth. He saw the need for positive self-regard as critical for such development. Therapy, in his view, was a way of “getting behind the mask”: dropping false fronts and becoming free and open to hidden aspects of one’s self. Characteristics of fully functioning persons included having an openness to experience, a trust in one’s own feelings, an internal locus of evaluation, a willingness to continually develop and become, a commitment to live each moment fully, a trustworthiness of human nature, and a greater richness and range of feelings. For both Maslow and Rogers, self-development was essentially a matter of removing impediments and deficiencies so that growth could naturally occur.

Allport (1961) formulated maturity as the individual’s unique potential for growth. He mentions work with patients, but underscores that his views evolved primarily through his own intellectual explorations. For him, motivation was the “go” of personality, in contrast to reactive views in behavioral psychology or psychoanalysis. Emphasis on drive satisfaction or sexual motives failed to capture aspects of competence, self-actualization, and ego autonomy. In his view, an adequate theory of motivation must focus on the present rather than unchanging motives forged in the past and must be pluralistic (allowing for many types of motives), including cognitive planning and intention. He saw individuals as seeking more than homeostasis (biological balance, satisfaction of drives). There are also proactive intentions, which he spoke of as functional autonomy. Allport acknowledged that psychology alone could not tell us what health, soundness, and maturity were, because ethical judgment was involved to some degree. His criteria of maturity included having strong interests outside the self, being capable of great intimacy with others, having emotional security and an accurate perception of reality, being self-objectified, which he defined as having insight, humor, and a unifying philosophy of life.

Jung (1933, 1965) and Von Franz (1964) wrote about individuation, offering a different formulation of growth and development. This vision evolved largely from his own self-examination and scrutinizing analyses of crises in his own life, though he noted parallels with some of his patients of a similar age. He considered individuation to be a natural, instinctive process, emphasizing that psychic growth could not be willed; it happens involuntarily and naturally, as manifest through one’s dreams. He saw the story of his life as the self-realization of the unconscious. This meant that individuation could not be approached purely as a scientific problem. Reason and rationalism set the boundaries too narrowly for us, he believed, leading to a pauperized individual. Jung’s personal experiences in middle age, and his observations of difficulties in patients of similar age, convinced him that the external achievements expected and rewarded by society were won at the cost of a diminution of personality. He felt there was a different meaning to the “afternoon of human life.” The significance of the morning of life was the development of the individual, the propagation of one’s kind, the care of children, the earning of money, and other external conquests. For him, money-making, social existence, family, and posterity were about the outward achievements of personality. Such things stood in the way of developing one’s inner self, which Jung saw as the meaning of psychology in the second half of life. In his words, “after having lavished its light upon the world, the sun withdraws it rays in order to illumine itself” (Jung, 1933, p. 109). Those who clung to the collective fears, beliefs, laws, and systems of the masses would not achieve self-realization in his view. Emphasis must be on exploring one’s inner self through dreams, fantasies, and creative imagination. Specific characteristics of individuation were not detailed, though emphasis was given to integrating conscious and unconscious aspects of one’s self, being in touch with the shadow (the dark side of the self), and accepting and expressing both masculine and feminine characteristics. The overall aim was to find harmony and balance in thinking, feeling, sensing, and intuiting.

Aristotle’s eudaimonia sits prominently in the background of all of the above efforts to articulate meanings and characteristics of personal growth (see Ryff & Singer, 2008). Written about in the *Nichomachean Ethics* (translated by Ross, 1925), Aristotle grappled with the fundamental questions of human existence: how should we live? Refusing to rely on superficial language (cant) or religious dogma, he sought a reasoned argument about what constitutes “the highest of all goods achievable by action?” He believed most people would answer happiness, but he saw notable differences in what is meant by happiness. To some, it is plain and obvious things like pleasure, wealth, or honor, but Aristotle dismissed those views as a life “suitable to beasts.” Instead, he defined the highest good as “activity of the soul in accordance with virtue” and then pushed himself to articulate the highest virtue. This is the essential message of eudaimonia, which he formulated as achieving the best that is within us. What Aristotle thus brought to personal growth is the idea of realizing one’s true and best nature, which is a lifelong endeavor. The task is to strive for self-realization, played out individually, each according to his or her own disposition and talents. Aristotle was concerned about action, not just abstract ideas: “we must become just by doing just acts.” These ideas will be revisited in the final section on future directions, where exemplars of personal growth will be considered with attention to their deeds and actions.

### 2.2. Defining and Measuring Personal Growth

Bringing the Ryff (1989) model to the empirical arena required creating definitions for each of the six components of well-being and then using these definitions to generate self-descriptive items to operationalize what it means to have (i.e., high scorer) or lack (i.e., low scorer) each dimension. Including both provides a guard against “response sets” such as the tendency to agree with all items, possibly because they have not been carefully read. Stated otherwise, the only way to obtain a high score for each dimension is to strongly agree with all the positively worded items and strongly disagree with all the negatively worded items.

Below are the definitions crafted for personal growth. They came from the preceding conceptual formulations.**Definitions of Personal Growth*****High scorer***Has a feeling of continued development; sees self as growing and expanding; is open to new experiences; has a sense of realizing his or her potential; sees improvement in self and behavior over time; is changing in ways that reflect more self-knowledge and effectiveness. Sample items:For me, life has been a continuous process of learning, changing, and growth.I think it is important to have new experiences that challenge how you think about yourself and the world.I have the sense that I have developed a lot as a person over time.***Low scorer***Has a sense of personal stagnation; lacks a sense of improvement or expansion over time; feels bored and uninterested with life; feels unable to develop new attitudes or behaviors. Sample items:When I think about it, I have not really improved much as a person over the years.I am not interested in activities that will expand my horizons.I do not enjoy being in new situations that require me to change my old familiar ways of doing things.

As detailed in Ryff (1989), large pools of items were written to operationalize each dimension of well-being and then subsequently evaluated via diverse psychometric criteria: (1) Did each item correlate more highly with its own scale than another scale? (2) Did the separate dimensions emerge as empirically distinct in factor analyses? (3) Were the scales empirically distinct from existing measures of well-being (discriminant validity)? (4) Did the scales show adequate test–retest reliability? These efforts provided the empirical foundation of the six different scales of well-being. Numerous subsequent studies examined the psychometric properties of this model (see Ryff, 2014), which has been translated into 40 different languages and is widely used around the world. Prior summaries of findings are available elsewhere (Ryff, 2018, 2024a; Ryff et al., 2021; Ryff & Singer, 2008). The overall model is not the focus herein; rather, interest is in one key dimension of well-being, namely, personal growth.

## 3. Scientific Findings on Personal Growth

It is important to note that the dimension of well-being receiving the greatest attention to date has been purpose in life, which has been found to predict longevity (Boyle et al., 2009; Cohen et al., 2016) as well as reduced risk for numerous health outcomes, such as myocardial infarction, stroke, and Alzheimer’s disease (E. S. Kim et al., 2013a, 2013b; Boyle et al., 2010), physical function and sleep disturbances (E. S. Kim et al., 2017; E. Kim et al., 2015), and drug use (E. S. Kim et al., 2020). This article, however, brings explicit attention to personal growth, arguably closest to Aristotle’s formulation of personal excellence and personal becoming. Summarized below are illustrative findings that have grown up around personal growth, beginning with links to demographic variables (e.g., age, educational status, race), and then moving to how personal growth correlates with diverse health outcomes and psychosocial factors. Much of the evidence assembled comes from a national longitudinal study of U.S. adults known as MIDUS (Midlife in the U.S) (www.midus.wisc.edu).

### 3.1. Demographic Correlates of Personal Growth

Recent findings from MIDUS (Boylan et al., 2025) document 20-year trajectories of all components of eudaimonic well-being. Personal growth emerges as uniquely important in showing the greatest within-person decline across three waves of assessment. Personal growth has thus been the most imperiled aspect of well-being over the past two decades. Such a decline is all the more notable, given selective attrition in the longitudinal sample across time (Radler & Ryff, 2010; Song et al., 2021). In addition, the 20-year trajectories showed significantly more decline in older compared to young and middle-aged adults, as well as in those with only a high school education compared to those with some college or a college degree. Stated otherwise, when examined longitudinally in a national sample of U.S. adults, personal growth shows the most dramatic decline across time, with older and less educated adults showing notable losses in sense of self-realization and personal becoming.

The above findings controlled for numerous factors, including gender, race, income, work status, and chronic conditions. It is worth noting, however, that distant findings from the MIDUS baseline assessments (Ryff et al., 2003) found, unexpectedly, that minority status was a *positive* predictor of all aspects of eudaimonic well-being compared to majority status. The title of the article thus asked: “Do the challenges of minority life hone purpose and growth?” Such a finding brought attention to eudaimonic well-being vis-à-vis existential challenges of living. Perceived discrimination was a consistent negative predictor of all EWB outcomes, particularly for women. The overall findings heighten the need to remember, per guidance from Frankl (1959/1992), that the existential aspects of purpose and growth can be strengthened as well as undermined by adversity and unjust experiences.

### 3.2. Personal Growth, Biomarkers, and Health

Multiple studies have examined how dimensions of eudaimonic well-being are linked with biomarkers and health. An early investigation using MIDUS data focused on links between educational status and the inflammatory marker, interleukin-6 (IL-6) (Morozink et al., 2010), which is implicated in multiple disease outcomes. Those with lower levels of education had higher levels of IL-6, thus replicating previous findings. However, some dimensions of eudaimonic well-being (i.e., environmental mastery, positive relations with others, purpose in life, self-acceptance) were found to moderate (buffer against) this effect. Personal growth did not emerge in these early findings as a significant protective factor.

In contrast, Boylan and Ryff (2015) examined the links between eudaimonic well-being and metabolic syndrome, measured with multiple risk factors, including central obesity, triglycerides, cholesterol, blood pressure, and fasting glucose. Findings showed that *only personal growth* and two hedonic indicators of well-being (life satisfaction, positive affect) were significant negative predictors of metabolic syndrome after adjusting for demographic and health covariates. Such findings brought notable attention to the beneficial effects of self-realization and personal becoming vis-à-vis multiple factors involved in metabolic syndrome. How these linkages come—what are the intervening pathways—requires further inquiry.

Recently, Cha et al. (2025) examined linkages between cumulative stress and epigenetic age in MIDUS, with dimensions of eudaimonic well-being again examined as possible moderators. Cellular epigenetic aging implicates a faster pace of biological aging relative to one’s chronological age and is predictive of morbidity and mortality. Findings from MIDUS showed that higher levels of cumulative stress were not directly predictive of epigenetic aging (measured with GrimAge2) but were contingent on levels of eudaimonia well-being (all six dimensions), such that those with low levels of well-being combined with high profiles of cumulative stress had significantly higher levels of epigenetic aging. Those with high levels of well-being (all dimensions) were protected against this outcome.

Other work has focused on the role of personal growth in the aftermath of cancer. Using MIDUS, Pudrovska (2010) tracked personal growth over 10 years among individuals with and without cancer. Decline was evident for both groups, consistent with prior findings showing age-related decline for personal growth and purpose in life. However, this study showed that the pace of decline was slower among those with cancer, suggesting that health challenges may be experiences of adversity that reduce, rather than enhance, the sense that one is experiencing less personal growth with aging.

Another realm of science has linked experiences of personal growth to cognitive functioning. The work complements prior studies (Boyle et al., 2010, 2012; Lewis et al., 2017) showing that purpose in life was linked with reduced risk for Alzheimer’s disease as well as better episodic memory and executive functioning. Toyama (2022) used multiple waves from MIDUS to show that personal growth predicted significantly smaller decreases in episodic memory over time, although it did not predict executive function. Britton et al. (2025) also used MIDUS longitudinal data to investigate cross-time links between multiple cognitive domains assessed by the Brief Test of Adult Cognition (BTACT) and different aspects of eudaimonic well-being. Using a BTACT composite score for overall cognition, findings showed that the strength of the longitudinal association between well-being and cognition varied across the dimensions: personal growth showed the strongest effects, followed by environmental mastery, and then self-acceptance and purpose in life. These findings underscore the importance of striving toward personal excellence as a positive influence on cognitive health.

Taken together, the above inquiries illustrate how personal growth, an aspect of eudaimonic well-being, has been linked with diverse aspects of health. Not all findings have been supportive. For example, as noted above, personal growth was not a significant protective factor in linking low educational status to a prominent inflammatory factor (IL-6). Alternatively, personal growth was the only aspect of eudaimonic well-being showing protection against metabolic syndrome. All aspects of eudaimonia, including personal growth, emerged as protective against the impact of cumulative stress on epigenetic age acceleration. Although personal growth is known to decline with aging, the pace of such decline is slower in cancer patients, suggesting that some health challenges may contribute to the perception that one is growing and changing over time. Although purpose in life eclipses other aspects of well-being in predicting reduced risk of cognitive decline, newer findings now show that personal growth predicts reduced decline in episodic memory over time as well as better-maintained cognitive functioning measured as a composite.

### 3.3. Personal Growth, Psychosocial Factors, and Interventions

Another line of inquiry has linked personal growth with psychosocial factors, seeking to advance understanding of what nurtures or facilitates growth experiences. Lee et al. (2018) observed that personal growth is largely thought of as intrapersonal (residing within the person), but interpersonal processes may be important in facilitating personal growth. Multiple studies, using data from MIDUS and a parallel study in Japan (MIDJA), were conducted to show that people’s perceptions of the supportiveness of their ties with close others predicted their levels of personal growth, with the effects mediated by self-confidence. Similarly, Toyama et al. (2020) examined a multitude of psychosocial factors, including social relationships, generativity, and spirituality, as influences on the personal growth of aging adults. Three waves of data from MIDUS were included, with findings showing that positive relationships with others become increasingly important for maintaining higher personal growth among older adults.

Eudaimonia has been used as a guide in intervention studies seeking to improve lives (Ruini & Ryff, 2016). Well-being therapy has been used to treat anxiety disorders in clinical contexts (Ruini & Fava, 2009) as well as in schools seeking to promote psychological well-being during adolescence (Ruini et al., 2009). At the other end of the life course, community-based interventions have been conducted with older adults, showing that the eudaimonic well-being is modifiable (Friedman et al., 2017, 2019). An 8-week program showed gains, especially in personal growth, positive relations, and self-acceptance. Importantly, eudaimonic well-being offers new directions in mental health practice, in contrast to decades of work focused primarily on mental illness and distress (Ryff, 2023a).

In sum, scientific findings about positive relationships with others, as well as intervention studies in early and later life, underscore that personal growth is malleable and can be enhanced. These are important messages for those who may lack a sense that they are making the most of their talents across time; evidence suggests the continued pursuit of personal excellence is, indeed, possible even in difficult life contexts.

## 4. Future Directions in Research and Practice Related to Personal Growth

Looking forward, the central task is to discern where science and practice need to go on the topic of personal growth, which is central to developmental, existential, and humanistic approaches to human well-being as well as Aristotle’s vision of eudaimonia. What follows advocates for three targeted future directions. First, there is the need to recognize that not all individuals are given equal opportunities to become their best selves; widening inequality is a growing impediment to self-realization among those lacking educational opportunities and adequate incomes. This reality implicates the growing problem of greed among advantaged segments of society. The second topic calls for greater emphasis on encounters with the arts and humanities as inputs that may help people to know and pursue their core life aspirations. The third direction advocates, per Aristotle’s message, for elevating virtue and ethics as key aspects of personal becoming. To bring these ideals into focus, the heroic actions of a few figures from human history who devoted themselves to overcoming social injustices are briefly examined.

### 4.1. Obstacles to Personal Becoming: The Costs of Widening Inequality

Sadly, growing evidence documents that experiences of eudaimonic well-being are increasingly sequestered among privileged segments of modern societies (Boylan et al., 2025; Ryff, 2023b, 2024c). Socioeconomic inequalities are also widening over time, especially in the U.S. (Kirsch & Ryff, 2016), such that top income earners have better educations, jobs, income, and wealth as well as a greater likelihood of stable marriages to successful partners; they also live in thriving neighborhoods and practice healthier lifestyles (Reeves, 2017). These problems have been exacerbated by major historical stressors, such as the Great Recession and the COVID-19 pandemic. Hardships of these wide-ranging events fell disproportionately on those who were already disadvantaged (Ryff, 2023b; Kirsch et al., 2023; Kirsch & Ryff, 2016).

Taken together, these disturbing realities make clear that opportunities to make the most of one’s talents and capacities—arguably the core of what personal excellence is about—are not equally distributed across the social order, but disproportionately accrue to those in privileged positions, defined primarily in terms of family background, education, and income. More troubling, additional work has shown that institutions of higher education are the primary means through which class hierarchies are perpetuated (see Ryff, 2026). That is, although higher education has long been thought to be a primary mechanism of upward mobility, recent empirical work shows that elite educational institutions are places where norms for financial gain are nurtured, i.e., where the pursuit of affluence, among those who already are affluent, is cultivated (Mendelberg et al., 2017). Desmond’s (2023) *Poverty, by America* further deepens understanding of this malaise via evidence that many are kept poor because others are profiting from their vulnerability.

Human history shows a longstanding concern with the problem of greed. The ancient Greeks believed greed violated norms of fairness and thereby contributed to civic strife (Balot, 2001). They called for public criticism and censure of greed. In the fourteenth century, Dante’s (1308/2006) *Divine Comedy* placed sins of greed and gluttony, along with fraud and dishonesty, in his nine circles of hell. In the eighteenth century, Smith’s (1776/1981) *Wealth of Nations* made the case for self-interest and capitalism, but even he recognized the problem of greed, distilled as the limitless appetites of the vain and insatiable. Research on health inequalities has grown in recent years, but most findings depict the stressors and health problems experienced by those in low SES positions (Marmot et al., 2008). Little attention has been given to those at the top, although psychologists have examined the dark side of the American dream, exemplified by the singular pursuit of financial success (Kasser & Ryan, 1993). Others have documented the greater sense of entitlement and narcissism among those with higher compared to lower class standing (Piff, 2014).

Going forward, spotlights are needed on these unequally distributed life opportunities that are preventing socioeconomically disadvantaged individuals from making the most of their talents and capacities. Efforts are needed by scientists, practitioners, and those involved in public policy regarding the allocation of government resources. A useful guide therein is Robeyns’ (2024) *Limitarianism*, which integrates ethics, political theory, economics, and public policy to explicate the case against extreme wealth, while calling for a better, fairer world.

### 4.2. Vital Inputs to Human Becoming: Encounters with the Arts and Humanities

Despite the presence of class hierarchies throughout human history, it is heartening that some working-class individuals found their way to great literature and philosophy, and thereby, became poets, artists, and writers. Examples are distilled in *A People’s History of Classics: Class and Greco-Roman Antiquity in Britain and Ireland from 1680 to 1939* (Hall & Stead, 2020). The book describes how working-class miners, readers, and poets engaged the ancient Greeks and Romans to expand their horizons, alleviate their boredom, and inform their politics:

“They used the Classics to prove their intellectual calibre, to express their plight and signal their consciousness of the class system; they also used it to subvert and undermine the authority of the classes that ruled them and to entertain themselves during their leisure hours”.

(p. 537)

Contemporary educators continue to argue for the arts and humanities as consequential realms for achieving self-realization and human potential (Nussbaum, 2010; Roth, 2014), albeit with less emphasis on alleviating class inequalities. Edmundson (2015) observes that our materialistic culture has lost the values and ideals needed by the human soul—courage, contemplation, and compassion—which are abundant in great literature. Bringing such work to his students, he repeatedly asks *Can you live it?* Does the work offer new or better ways of understanding the self and others? Does it point to alternative paths to a fulfilling life? Wordsworth’s poem, “Lines Composed a Few Miles Above Tintern Abbey” (1798), is offered as an example. Living in a din-filled city, the poet had a dull ache in his spirit, so he returned to a setting from his childhood that reminded him of the critical role of nature in his own vitality. Wordsworth’s poetry also served a vital function in the life of John Stuart Mill, who, despite a remarkable early education, found himself as a young adult without the happiness deemed central to utilitarian philosophy. He credited the poems for helping him recover from the crisis in his mental history (Mill, 1893/1989).

Edmundson (2004) sees the humanities as offering the best that has been known and thought, where “people are free to pursue their own hopes of becoming better than they are in a human sense—wiser, more vital, kinder, sadder, more thoughtful, more worth the admiration of their children” (p. 142). This is about taking ourselves to places where we learn about having more self-knowledge, fuller self-determination involving both mind and heart. Another prominent educator of great literature, Bloom (2000), emphasizes its role in strengthening the self, appreciating the ironic, and helping us prepare for change.

Importantly, great literature powerfully depicts societal problems, such as Dickens (1859/2004), *A Tale of Two Cities*, that brought horrors of the French Revolution—those imprisoned in the Bastille, the guillotine executions at the Place de La Concorde in Paris—to his readers. This blood bath of class retribution took more than 1200 lives, including the French Queen and King. Dickens distilled the context as follows: “…the frightful moral disorder born of unspeakable suffering, intolerable oppression, and heartless indifference” (p. 344). An example from contemporary fiction, *Call Me Zebra* (Van der Vliet Oloomi, 2018), tracks a family escaping from Iran on foot. They see themselves as anarchists, atheists, and autodidacts who take refuge in books. The family’s distilled philosophy was “love nothing except literature, the only magnanimous host there is in this decaying world” (p. 8). Memorization is key; sprinkled throughout the book are quotes from Nietzsche, Omar Khayyam, Dante, Goethe, Rilke, Kafka, Cervantes, Garcia Lorca, Dali, and Picasso.

Satire offers another powerful literary tool in the face of oppression and want. Swift’s (1729) *A Modest Proposal* suggested a solution for the poor of Ireland: their children should be well fed in their first years so they could be sent to England…. to be eaten, offering “a most delicious nourishing and wholesome food whether stewed, roasted, baked or boiled” (p. 3). The intent was to mock the heartless attitudes of the British toward the Irish via one of the greatest examples of sustained irony in the history of the English language. A contemporary example is *The Sellout* (Beatty, 2015), which covers race relations in the fictional town of Dickens, California, where poor and black Americans are left to fend for themselves. Sister cities are identified (Chernobyl, Juárez, Kinshasa), all known for their pollution, poverty, and dysfunction. The point is this: when faced with major societal ills, the arts and humanities are critical for awakening awareness of human suffering and mobilizing actions to alleviate it. Ideally, such awareness and action would be part of everyone’s personal becoming.

### 4.3. Return to Virtue and Ethics as Core Elements of Personal Becoming

Aristotle believed that the highest of all human goods was the activity of the soul in accord with virtue. In addition, he thought the highest virtue, eudaimonia, was about becoming one’s best self. It cannot be stressed enough that his view of personal excellence was singularly concerned with virtue and ethics. Potentiating the daimon within all of us requires having high moral standards and integrity. We cannot find our calling without engaging distinctions between what is right or wrong, good or bad, just or unjust, all demanding tasks.

One way to think about, if not inspire, eudaimonic becoming is to consider notable exemplars. The growing field of *Heroism Science* (Allison, 2016, 2024) is a place to find such individuals. Although heroic acts are often unexpected, short-term deeds, such as rescuing individuals from a burning building or a crashed plane, there is another enduring kind of heroism that involves persistent efforts, often over the course of one’s life, to address societal ills. Such enduring heroism in the face of inequality and injustice was recently examined (Ryff, 2024b) in the lives of notable individuals from human history. A subset of these cases is briefly distilled below. All confronted terrible injustices, and most were victims of unjust treatment themselves. Nonetheless, their journeys of personal becoming had a great impact on the lives of others.

Harriet Tubman (1822–1913) was born a slave and was beaten by her enslavers. She and her brothers escaped multiple times, traveling north to Pennsylvania. Eventually, she became a major American abolitionist and social activist (Clinton, 2004). Leading 13 such missions, she rescued many enslaved people through a network of safe houses known as the Underground Railroad. She led all the way to Canada. Tubman also worked as a cook and nurse for the Union army during the Civil War. Her hope was to become literate so that she could write her memoirs, but that aspiration was never achieved. Nonetheless, Harriet Tubman’s pursuit of her calling was responsible for saving the lives of many from the degradations of slavery.

Crazy Horse (1841–1877) was a member of the Ogalala Lakota Sioux nation who lived in present-day Wyoming (McMurtry, 2005). He took up arms against the U.S. government to fight against the encroachment of white settlers on Native American territories. After gold was discovered along the Bozeman Trail, General Sherman led the building of multiple forts in Sioux territory. Crazy Horse led attacks on these forts in the 1870s. The Battle of the Little Big Horn, where Custer’s forces went down to disastrous defeat, took place in 1876. Crazy Horse later surrendered, incapacitated by the harsh winters in which he and his people were starving. He died at age 35, stabbed by a soldier. Known for his courage, tenacity, and leadership, Crazy Horse fought to protect his people against impossible odds. In the Black Hills of South Dakota, land sacred for the Lakota, a giant monument was sculpted in his honor. The 2022 film “Lakota Nation vs. United States” reviewed 150 years of treaty violations with native Americans. The U.S. Supreme Court granted remuneration for the lost lands in 1980, but the Lakota people refused the money and continue to fight for the land itself. The heroic actions of Crazy Horse seeking to protect his people continue to inspire many.

Mohandas Gandhi (1869–1948) was raised in a Hindu family in coastal India. His companions were books; he entered an arranged marriage at age 13. After college and legal training, he spent 21 years in South Africa, where he faced severe discrimination. Returning to India in 1915, he assumed leadership of the Indian National Congress and began demanding independence from the British (Dalton, 2012). Gandhi led agitations related to famine, floods, and taxes, while railing against the Viceroy’s salary, over 5000 higher than the average salary in India. Thousands joined his salt march campaign and commitment to civil disobedience. His motto was “soul force and morality.” Gandhi’s fasting protests were credited with stopping religious riots and communal violence. Murdered by a Hindu nationalist, he was mourned around the world. His writings were simple and precise (Gandhi & Fischer, 2002), and his concept of nonviolence had a long history in Indian religious thought; it was viewed as the highest dharma (ethical value/virtue).

Malcolm X (1925–1965) was born Malcolm Little in Omaha, Nebraska, the 4th of 7 children (Marable, 2011). After his father’s death, viewed by some as a murder, and his mother’s hospitalization, he was moved to foster homes. Dropping out of high school, he began a life of drugs, gambling, and theft, which led to prison, where he began educating himself. After his release, he joined the Nation of Islam, changing his name to Malcolm X. As a preacher, he was a skilled speaker, humorous, and charismatic. Malcolm X fought against segregation and was critical of the civil rights movement. He broke with the Nation of Islam after discovering the sexual misconduct of Elijah Muhammad, who was impregnating young girls serving as his secretaries. Malcolm then received death threats and was assassinated in 1965 while delivering a speech at Harlem’s Audubon Ballroom. He is credited with raising the self-esteem of Black Americans and reconnecting them with their African heritage. Some believed he better articulated the fundamental problems of inequality than the civil rights movement. He was eulogized (Davis, 1965) for his decency, humanity, and warmth.

What can be learned from this brief look at widely known heroes? A first observation is that most transformed their own experiences of grave injustices into a virtuous calling to help other oppressed individuals. Being on the receiving end of brutal mistreatment likely shaped their courage and resolution to effect significant societal change. Second, all were deeply aspirational, i.e., motivated to accomplish something significant and lasting with their own lives. Many strived to educate themselves along the way. Third, they differed notably in whether their life pursuits (eudaimonic becoming) worked within extant social systems and norms to effect change, or involved dramatic, even violent, activities to defeat their oppressors. Finally, together they embodied the vision, purpose, and commitment required by those who choose to devote their lives to challenging discrimination and injustice in hopes of making the world a better place. This kind of enduring heroism is greatly needed in our own era.

## 5. Summary

Extensive territory has been covered in this article, written for a Special Issue of *Behavioral Sciences* on the topic of personal growth. The opening segment examined conceptual underpinnings of personal growth in a widely used model of eudaimonic well-being (Ryff, 1989). The intent was to convey the wide array of writings from the middle of the last century on the motivation for, and enactment of, the realization of one’s full potential, i.e., what Aristotle described as personal excellence in depicting the highest of all human goods. Ideas from these many sources were integrated into a formal definition of personal growth from which self-report instruments were constructed to quantitatively measure who does and does not have high levels of this aspect of psychological well-being.

The next section reviewed diverse scientific findings that have grown up around this operationalization of personal growth. Using data from the MIDUS national longitudinal study of U.S. adults, findings have shown that personal growth tends to decline with aging and is lower among those with less education. Recent work on 20-year trajectories documents that personal growth is the most imperiled aspect of psychological well-being across time. Other findings have shown the health benefits of having higher levels of personal growth, including lower biological risk factors and less epigenetic (fast-paced) aging, as well as better memory and cognitive functioning. Supportive social relationships have also been found to predict higher levels of personal growth across time. Interventions in clinical contexts as well as in schools for adolescents and community centers for older adults show that personal growth, along with other aspects of eudaimonic well-being, is modifiable.

Seeking to activate new directions in research and practice, the final section distilled three topics. The first emphasized problems of widening inequality, which are undermining the opportunities for many, particularly socioeconomically disadvantaged segments of modern societies, to make the most of their talents and capacities over time. This is a notable problem driven, in part, by heightened greed among some who are privileged, a longstanding concern throughout human history. The call is for scientists, practitioners, and those involved in the public policy arena to rein in extreme wealth so as to facilitate a fairer world that nurtures the human becoming, the personal growth, of all.

The second direction advocated for encounters with the arts and humanities as critical inputs needed for making the most of personal talents. Examples were provided from notable teachers of great literature who understood its powerful role in strengthening the self, fostering courage, and the contemplation needed to be one’s best self. The arts further inform us in powerful and poignant ways about societal ills and human suffering. Examples from literary classics as well as recently acclaimed works of fiction, including satire, were included to illustrate the transformational power of great art.

The final future direction called for a return to virtue and ethics as core elements in thinking about personal becoming. The central message is that without moral standards and integrity, enactments of personal becoming can become harmful to self, others, and even society. Bringing specificity to these challenges, select exemplars from human history were put forth. All of these individuals led deeply aspirational lives focused on righting human wrongs, striving to make the world a more just place, particularly among those who have been historically oppressed. The point of this section was thus to elevate the heroic side of personal growth, wherein achieving one’s daimon is profoundly wrapped up with striving to make the world a better place.

## Data Availability

No new data were created or analyzed in this study.
